# Glutathione and Bcl-2 targeting facilitates elimination by chemoradiotherapy of human A375 melanoma xenografts overexpressing *bcl-xl*, *bcl-2*, and *mcl-1*

**DOI:** 10.1186/1479-5876-10-8

**Published:** 2012-01-10

**Authors:** Salvador Mena, María L Rodriguez, Angel Ortega, Sonia Priego, Elena Obrador, Miguel Asensi, Ignacio Petschen, Miguel Cerdá, Bob D Brown, José M Estrela

**Affiliations:** 1 Department of Physiology, University of Valencia (Valencia, Spain; 2 Radiotherapy Service, La Fe Hospital (Valencia, Spain; 3 Department of Pathology, University of Valencia (Valencia, Spain; 4 Formerly at Genta Incorporated, Berkeley Heights, New Jersey (USA; 5 Dicerna Pharmaceuticals Watertown, Massachusetts (USA

## Abstract

**Background:**

Bcl-2 is believed to contribute to melanoma chemoresistance. However, expression of Bcl-2 proteins may be different among melanomas. Thus correlations among expression of Bcl-2-related proteins and *in vivo *melanoma progression, and resistance to combination therapies, was investigated.

**Methods:**

Human A375 melanoma was injected s.c. into immunodeficient nude mice. Protein expression was studied in tumor samples obtained by laser microdisection. Transfection of siRNA or ectopic overexpression were applied to manipulate proteins which are up- or down-regulated, preferentially, during melanoma progression. Anti-*bcl*-2 antisense oligonucleotides and chemoradiotherapy (glutathione-depleting agents, paclitaxel protein-binding particles, daunorubicin, X rays) were administered in combination.

**Results:**

*In vivo *A375 cells down-regulated pro-apoptotic *bax *expression; and up-regulated anti-apoptotic *bcl-2*, *bcl-xl*, and *mcl-1*, however only Bcl-2 appeared critical for long-term tumor cell survival and progression *in vivo*. Reduction of Bcl-2, combined with partial therapies, decreased melanoma growth. But only Bcl-2 targeting plus the full combination of chemoradiotherapy eradicated A375 melanoma, and led to long-term survival (> 120 days) without recurrence in 80% of mice. Tumor regression was not due to immune stimulation. Hematology and clinical chemistry data were within accepted clinical toxicities.

**Conclusion:**

Strategies to target Bcl-2, may increase the effectiveness of antitumor therapies against melanomas overexpressing Bcl-2 and likely other Bcl-2-related antiapoptotic proteins.

## Background

Malignant melanoma is one of the most highly invasive tumors, and its mortality rates have been rapidly increasing above those of any other cancer in recent years. Surgical resection and systemic chemotherapy are still the main therapeutic [1]. However systemic chemotherapy faces two major problems: tumor resistance and toxicity towards normal tissues [2].

Bcl-2 plays a major role in preventing apoptosis and has been linked to chemotherapy resistance in melanoma [3-5]. Bcl-2 also appears able to inhibit e.g. beclin 1-dependent autophagic cell death, which is a nonapoptotic pathway [6]. However there are striking inconsistencies for the expression of Bcl-2 family proteins with melanoma progression, particularly for Bcl-2. Roughly one-third of all available data suggests an increase in *bcl*-2 expression with advancing melanoma, while another third suggests a decrease [7-9]. The general consensus is that pro-apoptotic Bax is decreased with melanoma progression while anti-apoptotic Bcl-xl appears to increase [7]. Despite these controversies it has been shown that when xenografted into severe combined immunodeficient mice, 518A2 human melanoma cells with silenced *bcl*-2 either failed to grow at all or grew to tumors of low volume and then completely regressed; whereas control cells with normal levels of Bcl-2 protein expanded to be large, necrotic tumors [10]. Besides, we reported that G3139 (oblimersen sodium, a *bcl*-2-AS that selectively targets *bcl*-2 RNA for degradation and decreases Bcl-2 protein; Genta Inc.), if combined with a glutathione (GSH) depleting strategy, facilitates elimination of murine B16-F10 melanoma liver metastases by chemoradiotherapy and biotherapy [11]. These facts suggest that Bcl-2 may be an important target. Nevertheless, whether Bcl-2 is a key target against malignant melanoma, as well as the relationship among expression of Bcl-2-related proteins, *in vivo *melanoma growth, and resistance to therapy are still open questions with important clinical implications. We used human A375 melanoma xenografts, widely used as an experimental model [12], to investigate these questions. Our results show that, although *in vivo *growing A375 melanoma cells preferentially overexpress different Bcl-2-related proteins (as compared to cultured A375 cells), *in vivo *targeting of Bcl-2 leads to growth inhibition and sensitization to combined chemoradiotherapy.

## Materials and methods

### Cell culture

A375 human melanoma was from the American Type Culture Collection. A375 cells were grown in Dulbecco's modified Eagle's medium (DMEM) (Invitrogen, San Diego, CA), pH 7.4, supplemented with 10% FCS (Biochrom KG, Berlin, Germany), 100 units/ml penicillin and 100 μg/ml streptomycin. Cultures were maintained at 37°C in a humidified atmosphere with 5% CO_2_. Cells were harvested by incubation for 5 min with 0.05% (w/v) trypsin (Sigma, St. Louis, MO) in PBS (10 mM sodium phosphate, 4 mM KCl, 137 mM NaCl), pH 7.4, containing 0.3 mM EDTA, followed by the addition of 10% FCS to inactivate the trypsin. Cell numbers were determined using a Coulter Counter (Coulter Electronic Inc., Miami, FL). Cellular viability was assessed as previously reported [11], by measuring trypan blue exclusion and leakage of lactate dehydrogenase activity.

### Transfection of red fluorescent protein

The pDsRed-2 vector (Clontech Laboratories Inc., Palo Alto, CA) was used to engineer A375 clones stably expressing red fluorescent protein (RFP). This vector expresses RFP and the neomycin resistance gene on the same bicistronic message. Cultured A375 cells were transfected before reaching confluency. Transfection of the pDsRed-2 vector was carried out using linear 25 kDa polyethylenimine (PEI) (PolySciences, Inc., Warrington, PA), as described for adherent cell lines by the manufacturer. Cells were incubated for 4 h with the PEI-DNA complex in 5% of their initial culture medium (DMEM containing 10% fetal calf serum) volume. After that 4-h period the culture medium volume was restored to 100%, and cells were harvested 4 days after transfection. Cells were then harvested (as above) and subcultured into selective medium that contained 200 μg/ml of geneticin (Invitrogen). The level of geneticin was increased to 2,000 μg/ml stepwise. High-Performance Cell Sorting (DAKO, Copenhagen, Denmark) was used to select geneticin-resistant A375 clones expressing the RFP (A375-RFP) and showing high fluorescence emission. These cells were seeded in 96 wells plates, and their growth was followed by immune-fluorescence microscopy to select clones showing stable fluorescence emission.

### Animals and diets

Female *nu/nu *nude mice (6-8 weeks; Charles Rivers Laboratories, Wilmington, MA) were fed *ad libitum *on a standard diet or an equivalent diet but glutamine (Gln)-enriched diet (GED, 15% of total dietary nitrogen from Gln; Harlan Teklad Animal Diets & Bedding, Madison, WI [11]. Both diets were isonitrogenous and isocaloric. Mice were kept on a 12-h-light/12-h-dark cycle with the room temperature at 22°C. Procedures involving animals were in compliance with international laws and policies (EEC Directive 86/609, OJ L 358. 1, December 12, 1987; and NIH Guide for the Care and Use of Laboratory Animals, NIH Publ. No. 85-23, 1985).

### Tumor xenografts

For cancer cell xenograft experiments, *nu/nu *nude mice were inoculated s.c. (foot-pad) with 10 × 10^6 ^A375 or A375-RFP cells per mouse. Tumor volume was calculated based on two dimensions, measured using calipers, and was expressed in mm^3 ^according to V = 0.5a × b^2^, where a and b are the long and the short diameters of the tumor, respectively. For histological analysis xenograft samples were fixed in 4% formaldehyde, paraffin embedded, and stained with hematoxilin & eosin and safran. Mice were monitored after inoculation, and tumor measurements were taken every 2 days.

### Isolation and compartmentation of A375-RFP cells

Cell dispersion was carried out in minced tumor tissue as follows: 1) trypsinization (25 mg of fresh tissue per milliliter in Mg^2+ ^and Ca^2+ ^free PBS supplemented with 0.2% trypsin plus 0.5 mM EDTA plus 5 mM glucose, 3 min at 37°C); 2) three washes in PBS; 3) collagenase digestion (in PBS supplemented with 0.5 mg of collagenase/ml plus 5 mM glucose, 5 min at 37°C) (steps 1 and 3 were performed in Erlenmeyer flasks where the gas atmosphere was O_2_/CO_2_, 19:1). Then cells were washed three times in PBS and resuspended in 1 ml of ice-cold PBS, filtered through a 44-μm pore mesh and analyzed using a MoFlo High-Performance Cell Sorter (DAKO). Fluorescent A375 cells were separately gated for cell sorting and collected into culture chambered slides (Nalge Nunc International Corp., Naperville, IL), and harvested and plated in 25-cm^2 ^polystyrene flasks (Falcon Labware). Rapid separation of cytosolic and mitochondrial compartments was as previously described [13].

### Laser microdissection

Excised A375-RFP tumor samples were embedded in freezing medium OCT (Tissue-Tek, Electron Microscopy Sciences, Hatfield, PA) and flash-frozen using isopentane and following Leica Microsystems' (Wetzlar, Germany) instructions to preserve RNA. Five-μm tissue slices were obtained using a Leica 2800E Frigocut Cryostat Microtome. Tumor cells were separated using a Leica LMD6000 Laser Microdissection System equipped with an automated fluorescence module.

### RT-PCR and detection of mRNA

Total RNA was isolated using the trizol kit from Invitrogen and following manufacturer's instructions. cDNA was obtained using a random hexamer primer and a MultiScribe Reverse Transcriptase kit as described by the manufacturer (TaqMan RT Reagents, Applied Biosystems, Foster City, CA). A PCR master mix and AmpliTaq Gold DNA polymerase (Applied Biosystems) were then added containing the specific primers (Sigma-Genosys):

*bax *(F-CCAGCTGCCTTGGACTGT, R-ACCCCCTCAAGACCACTCTT);

*bak *(F-TGAAAAATGGCTTCGGGGCAAGGC, R-TCATGATTTGAAGAATCTTCGTACC);

*bad *(F-AGGGCTGACCCAGATTCC, R-GTGACGCAACGGTTAAACCT);

*bid *(F-GCTTCCAGTGTAGACGGAGC, R-GTGCAGATTCATGTGTGGATG);

*bik *(F-ATTTCATGAGGTGCCTGGAG, R-GGCTTCCAATCAAGCTTCTG);

*bim *(F-GCCCCTACCTCCCTACAGAC, R-CAGGTTCCTCCTGAGACTGC);

*bcl-*2 (F-CTCGTCGCTACCGTCGTGACTTCG, R-CAGATGCCGGTTCAGGTACTCAGTC);

*bcl-w *(F-GGTGGCAGACTTTGTAGGTT, R-GTGGTTCCATCTCCTTGTTG);

*bcl-xl *(F-GTAAACTGGGGTCGCATTGT, R-TGGATCCAAGGCTCTAGGTG);

*mcl-*1 (F-GAAAGCTGCATCGAACCATT, R- ACATTCCTGATGCCACCTTC);

glyceraldehyde-3P-dehydrogenase (GAPDH) (F-CCTGGAGAAACCTGCCAAGTATG, R-GGTCCTCAGTGTAGCCCAAGATG).

Real-time quantitation of the mRNA relative to GAPDH was performed with a SYBR Green I assay, and a iCycler detection system (Biorad, Hercules, CA). Target cDNA was amplified as follows: 10 min at 95°C, then 40 cycles of amplification (denaturation at 95°C for 30 sec and annealing and extension at 60°C for 1 min per cycle). The increase in fluorescence was measured in real time during the extension step. The threshold cycle (C_T_) was determined, and then the relative gene expression was expressed as: fold change = 2^-Δ(Δ C^_T_^) ^, where Δ C_T _= C_T _target - C_T _GAPDH, and Δ (Δ C_T_) = Δ C_T _treated - Δ C_T _control [14].

### Transfection of small interfering RNA and stable short hairpin interfering RNA constructs

The PSilencer 3.1-H1 linear vector from Ambion (Austin, TX) was used to obtain long-term gene silencing. The *bcl*-2, *bcl-xl*, and *mcl*-1siRNA inserts had sense and antisense sequences: *bcl-xl *sense 5'-CAGGGACAGCATATCAGAG-3' and antisense 5'-CTCTGATATGCTGTCCCTG-3'; *bcl*-2 sense 5'-AGTACATCCATTATAAGCT-3' and antisense 5'-AGCTTATAATGGATGTACT-3'; *mcl*-1 sense 5'-AAGUAUCACAGACGUUCUCTT-3' and antisense 5'-GAGAACGUCUGUGAUACUUTT-3'. The negative control vector, that expresses a hairpin siRNA with limited homology to any known sequences in human, was provided by the vector kit (Ambion). Recombinant PSilencer 3.1-H1 vector was transformed into competent E. coli DH5α (Takara Bio Inc., Shiga, Japan), according to the supplier protocol, and then bacteria were cultured, harvested, centrifuged, and subjected to SDS-alkaline lysis following standard methods (http://www.cshprotocols.org). Endotoxins were removed from the lysate by simple extraction-phase separation steps. The plasmid DNA was further purified by adsorption onto silica using GenElute Endotoxin-free Plasmid Maxiprep Kit (Sigma). The purified DNA was diluted to 1 mg/mL and frozen at -20°C. Transfections with the pSH1-*bcl-xl*, pSH1-*bcl*-2, or pSH1-*mcl*-1 plasmids were performed using a standard lipofection method (http://www.cshprotocols.org). Stably transfected clones were selected in medium containing 0.5 mg/mL geneticin (Invitrogen). Established clones were grown in medium supplemented with 10% FCS and 0.5 mg/mL geneticin. Silencing was confirmed by immunoblotting.

The psiRNA-h7SK kit from Invivogen (San Diego, CA), containing the psiRNA vector expressing shRNA targeting human *bcl*-2 gene, was used to generate anti-*bcl*-2 shRNA and for cell transfection.

Based on the design principles for shRNA constructs, we selected RNAi target sites within the open reading frame of human *bcl-xl *and *mcl*-1. The specific base sequences of the target sites of *bcl-xl *and *mcl*-1 were 5'-GGAGAT-GCAGGTATTGGTGAG-3' and 5'-ACGCGGUAAUCGGACUCA A-3', respectively. For each target sequences, a pair of sense and anti-sense strands was designed; their respective complementary chains were then synthesized by annealing. The negative control plasmid vector HK sequence 5'-GACTTCATAAGGCGCATGC-3' was used as a control (it does not target any specific human gene).

Lentiviral infections were done following standard methodology (Cell Biolabs, San Diego, CA), and the potency and specificity of each construct were determined by protein immunoblotting.

### *bax *gene transfer and analysis

The Tet-off gene expression system (Clontech, Palo Alto, CA) was used to insert the human *bax *gene and for transfection into A375 cells following manufacturer's instructions. Bax protein was detected by western blot (see above) using monoclonal antibodies anti-human Bax (Santa Cruz Biotechnology).

### Western blot analysis

Cultured cells, harvested as indicated above, or finely minced tissues were washed twice in ice-cold Krebs-Henseleit bicarbonate medium (pH 7.4). Cell or tissue extracts were made by freeze-thaw cycles (cells) or homogenization (tissues) in a buffer containing 150 mM NaCl, 1 mM EDTA, 10 mM Tris-HCl, 1 mM phenylmethylsulfonyl fluoride, 1 μg/ml leupeptin, 1 μg/ml aprotinin, and 1 μg/ml pepstatin, pH 7.4. The extracts were centrifuged at 10,000 × g for 30 min. Cell/tissue lysate supernatants were separated for protein determination. All steps were performed at 4°C. Fifty μg of protein (as determined by the Bradford assay [15]) were boiled in Laemmli buffer and resolved by 12.0% SDS-PAGE. Proteins were transferred to a nitrocellulose membrane and subjected to western blotting using mouse IgG1 monoclonal antibodies raised against human Bcl-2 or Bcl-xl (Cell Signaling Technology, Danvers, MA). Blots were developed using horseradish peroxidase-conjugated secondary antibody and enhanced chemiluminescence (ECL system, GE Healthcare, Piscataway, NJ).

### Antisense oligonucleotides

Fully phosphorothioate 18-mer *bcl-*2-AS were from Genta Incorporated (Berkeley Heights, NJ). G3139 (human; sequence: 5'-TCTCCCAGCGTGCGCCAT-3'), G3622 (reversed G3139 sequence control: 5'-TACCGCGTGCGACCCTCT-3'), and G4243 (G3139 labeled by incorporation of a 5-carboxyfluorescein nucleotide at the 5' end).

### Analysis of Bcl-2 levels

Bcl-2 protein was analysed by western blotting, and quantitated in the soluble cell/tissue fraction by enzyme immunoassay [14] using a monoclonal antibody-based assay from Sigma (St. Louis, MO) (one unit of Bcl-2 represents the amount of Bcl-2 protein in 1000 non-transfected A375 cells).

### GSH measurement

GSH in tumor and non-tumor tissues was determined, following procedures previously described [16], by liquid chromatography-mass spectrometry using a Quattro micro triple-quadrupole mass spectrometer (Micromass, Manchester, UK) equipped with a Shimadzu LC-10AD*VP *pump and SCL-10A*VP *controller system with an SIL-10AD*VP *autoinjector (Shimadzu Corporation, Kyoto, Japan). Tissue/blood sample collection and processing were performed according to published methodology, where rapid N-ethylmaleimide derivatization is used to prevent GSH auto-oxidation [17].

### Irradiation procedure

X rays were administered using a 6 KeV SL75 linear accelerator from Philips. Each mouse was anesthetized with nembutal (50 mg/kg i.p.), and fixed on a Perspex platform. Radiotherapy was administered at a rate of 2.0 Gy/min. The radiation beam was focused only on the tumor. The irradiated area was fixed to a maximum of 1.5 cm^2^, and the rest of the mouse had lead protection.

### Evaluation of therapy-induced *in vivo *toxicity

This included the following parameters: animal weight, complete blood cell count, and standard blood chemistry.

### Serum cytokines

Blood samples were centrifuged at 14,000 rpm for 10 min at 4°C to separate the serum. Concentrations of IL-1α, IL-1β, IL-2, IL-6, IL-10, IL-12, TNF-α, and IFN-γ in the serum was determined using mouse cytokine ELISA kits from Innovative Research (Novi, MI); whereas IFN-α was measured using a kit from Antigenix Station (New York, NY). Manufacturer's protocol was followed in all cases. Results were read with a Dynex MRXII ELISA reader (ThermoLabsystems, Chantilly, VA). Quantification of secreted cytokines was accomplished by normalization of the ELISA data with a standard cytokine dose curve.

### Statistical significance

Data were analyzed by Student's t test.

## Results

### Expression of Bcl-2 family proteins in A375 melanoma *in vivo*

**Figure 1:**
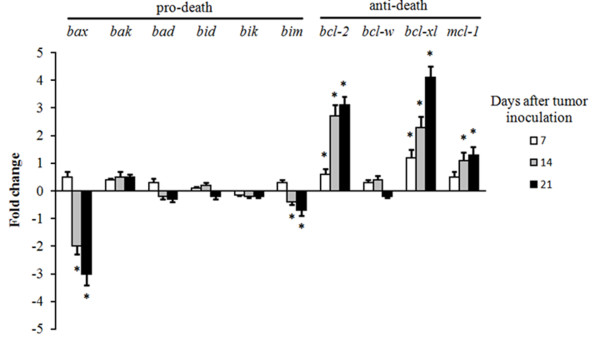
**Expression of pro-death and anti-death Bcl-2 genes in A375 melanoma xenografts**. A375-RFP cells were isolated by laser microdissection (as indicated under Methods) 7, 14, and 21 days after tumor inoculation. The data, expressing fold change (quantitative RT-PCR, see under Methods for calculations), show mean values ± S.D. for 5-6 different experiments (*p < 0.05 for all genes displayed comparing A375-RFP cells isolated from ***in vivo ***growing tumors versus 24 h-cultured A375-RFP cells). No significant differences in expression of Bcl-2-related genes when control A375 and A375-RFP cells were compared ***in vitro ***(not shown).

Our first task was to investigate if the expression of Bcl-2 and related proteins affecting apoptosis changes during melanoma growth *in vivo *relative to growth *in vitro*. As compared to cultured *in vitro *controls, A375 cells growing *in vivo *significantly down-regulate expression of pro-apoptotic *bax*; and up-regulate anti-apoptotic *bcl*-2, *bcl-xl *and *mcl*-1 (Figure 1). Thus suggesting that A375 growth *in vivo *is associated with activation of pro-survival mechanisms in the cancer cells. The decrease in *bax *and the increase in *bcl-xl *expression were in agreement with previous results where these changes were initially associated with clinical progression of melanoma [7]. However, it was still unclear whether the decrease in the pro-death Bax and/or the increase in the anti-death Bcl-2, Bcl-xl, and Mcl-1 proteins are merely a defence response against immune and microenvironment challenges, or were necessary for *in vivo *melanoma progression. In order to answer this question, mice were inoculated with A375 cells transfected with the Bax gen (A375/Tet-*bax*), or with siRNA specific for Bcl-2 (A375/*bcl*-2-siRNA), Bcl-xl (A375/*bcl-xl*-siRNA), or Mcl-1 (A375/*mcl*-1-siRNA).

### Specific *bcl-xl *or *bcl-2 *silencing inhibits *in vivo *A375 melanoma growth

**Figure 2:**
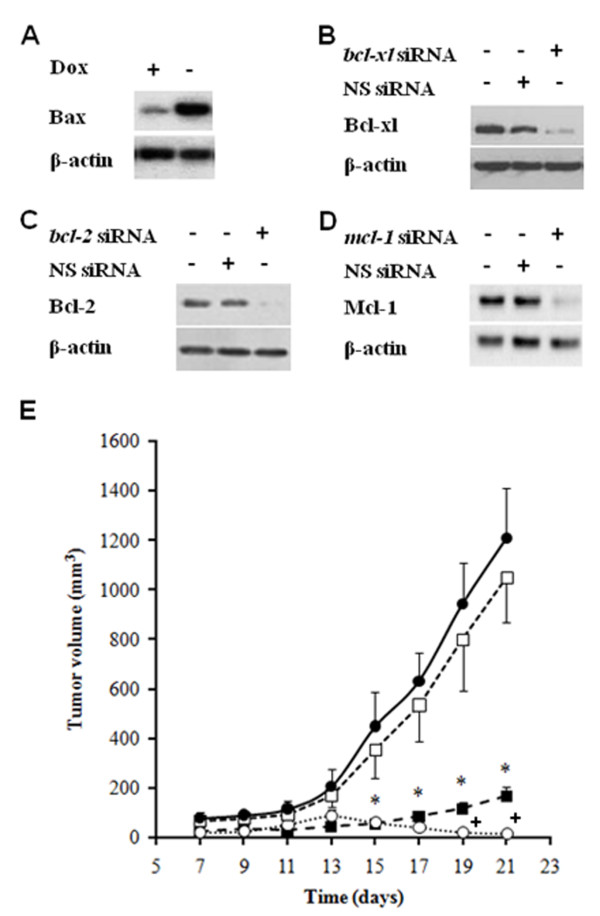
**Effect of shRNA-induced *bcl-xl*, *bcl*-2, or *mcl*-1 silencing and of ectopic *bax *overexpression on A375 xenograft growth**. siRNA-induced ***bcl-xl***, ***bcl***-2, or ***mcl***-1 silencing was used for preliminary ***in vitro ***experiments, whereas cells stably expressing shRNAs were used for ***in vivo ***experiments (see under Methods). (A) western blots of Bax in A375 and A375/Tet-***bax ***cells. (B, C, D) western blots of Bcl-xl, Bcl-2, and Mcl-1, respectively, in A375 cells transfected with ***bcl-xl ***siRNA, ***bcl***-2 siRNA, ***mcl***-1 siRNA, or non-specific (NS) siRNA. (E) ***in vivo ***growth of A375 (●), A375/***bcl-xl***-shRNA (■), A375/***bcl***-2-shRNA (○), and A375/Tet-***bax ***cells (□). Western blots correspond to cultured transfected cells, and are similar to those obtained in extracts of xenografts (see Methods) 1 or 2 weeks after tumor inoculation (not shown), thus indicating stability of each plasmid expression. Tumor growth (E) was measured during a 3-week period. Data are means ± S.D. of 9-10 mice per group. The significant test refers, for all groups (E), to the comparison between A375/***bcl-xl***-shRNA, A375/***bcl***-2-shRNA, or A375/Tet-***bax ***cells and A375 controls (*p < 0.01). A375/***bcl***-2-shRNA was also compared versus the A375/***bcl-xl***-shRNA group (**^+^**p < 0.01) (E). Rates of tumor growth obtained with A375/***mcl***-1-shRNA cells were not significantly different from those displayed for A375/Tet-***bax ***cells (□) (E) (not shown). Rates of tumor growth obtained with A375 cells transfected with empty Tet-off vector or with NS shRNA were not significantly different from those displayed for untransfected A375 controls (●) (E) (not shown).

After inoculation, control A375 xenografts grow exponentially. Ectopic *bax *overexpression (Figure 2A) did not affect A375 growth as compared to controls (Figure 2E). Specific siRNA-induced silencing of *mcl*-1 (Figure 2D) did not affect significantly melanoma growth (Figure 2E). Specific siRNA-induced silencing of either *bcl-xl *(Figure 2B) or *bcl*-2 (Figure 2C) sharply decreased melanoma growth (Figure 2E), but the involvement of these two anti-apoptotic gene family members was not equivalent. While A375/*bcl-xl*-siRNA tumors tended to progress slowly (167*±*38 mm^3^, approximately 13.8% of the tumor volume calculated for control A375 tumors 3 weeks after inoculation), A375/*bcl*-2-siRNA tumors tended to regress (86 ± 25 mm^3 ^1 week after inoculation, and < 20 mm^3 ^3 weeks after inoculation) (Figure 2E) (expression levels of the relevant apoptosis-related proteins in the tumors assessed in Figure 2E are presented in Additional file 1 Table S1). Therefore, despite the changes in gene expression displayed in Figure 1, *in vivo *A375 melanoma growth appears highly dependent on Bcl-2, whereas Bcl-xl and Mcl-1 expression did not substitute for Bcl-2. Our next task was to test if Bcl-2 represents a key therapeutic target to reduce melanoma cell survival *in vivo*.

**Table 1:** Effect of Bcl-2 and GSH depletion on the *in vivo *response of A375 melanoma to chemoradiotherapy.

	Tumor volume measurement (mm^3^)					
	
Days after treatment		1			15	
***Treatment***	**PS**	**G3139**	**G3139+ACV+VRP**	**PS**	**G3139**	**G3139+ACV+VRP**
	
**None**	192 ± 59	138 ± 28	175 ± 61	807 ± 240	953 ± 330	618 ± 265
**PAC.PBP**	153 ± 61	97 ± 31	74 ± 30*^+^	> 1000	731 ± 148	547 ± 138
**DNR**	220 ± 70	156 ± 49	117 ± 44*	> 1000	> 1000	> 1000
**PAC.PBP + DNR**	83 ± 26^++^	66 ± 18^++^	45 ± 12**^++^	> 1000	488 ± 177	352 ± 95
**X rays**	263 ± 88	84 ± 26**^+^	71 ± 25**^++^	> 1000	769 ± 260	641 ± 189
**X rays + PAC.PBP**	150 ± 44	93 ± 15*^+^	105 ± 38	643 ± 167	161 ± 75**^++^	96 ± 36**^++^
**X rays + DNR**	246 ± 71	96 ± 36**	53 ± 9**^++^	926 ± 225	694 ± 166	502 ± 190**
**X rays + PAC.PBP + DNR**	42 ± 14^++^	< 20	< 20	514 ± 116^+^	43 ± 12**^++^	N.D.

The treatment regimen is indicated in the scheme placed above the table. Mice were fed *ad libitum *on a GED (see Methods) starting 1 week before tumor inoculation. Verapamil (VRP, 1 mg/kg) and acivicin (ACV, 2 mg/kg) were given i.p. daily (1 dose × day) starting 7 days after tumor cell inoculation (days 7 to 16). G3139 (20 mg/kg) was given i.v. on days 7, 10, 13, and 16. Paclitaxel protein-binding particles [PAC.PBP, 32.5 mg/kg (human MTD × 5)] was given i.v. on days 7 and 10. Daunorubicin [DNR, 3.125 mg/kg (human MTD × 2.5)] was given i.v. on days 13 and 16. Animal doses of chemotherapy were calculated using NCI guidelines (www.cancer.gov) and the conversion factor for mice published by the FDA (www.fda.gov). Mice received fractionated X-ray therapy (15 Gy/day focused on the tumor) on days 8 and 9. Total X-ray dose was selected, as effective and safe, after testing a range of 5 to 40 Gy in the absence or in the presence of the full combined therapy (not shown). Mice were sacrificed when tumor volume was > 1000 mm^3^. Tumor size on day 7 after inoculation was, in all cases, of 50-75 mm^3^. Body weight of mice treated with the full combination was approximately 75% and 83% of controls (treated with physiological saline) 1 or 15 days, respectively, after the treatment. Data are means ± S.D. for 18 to 20 different mice. N.D., nondetectable. Histologic examination (see Methods) confirmed that, in 16 of 20 (80%) mice, the full treatment achieved a complete tumor regression. *P < 0.05, **P < 0.01 comparing for each condition G3139- or G3139 + VRP + ACV-treated tumor-bearing mice versus treatment with physiologic saline. ^+^P < 0.05, ^++^P < 0.01 comparing, in mice treated with physiologic saline, G3139, or G3139 + VRP + ACV, all experimental conditions versus none under treatment.

### Tumor Bcl-2 depletion *in vivo*

In a previous report the *bcl*-2-AS G3139 was found effective in depleting selectively Bcl-2 in metastatic B16- F10 melanoma cells [11]. Recently, periodic G3139 monotherapy (high dose, i.v., Q2-3 days) yielded superior antitumor efficacy (including different experimental models) when compared with low-dose daily i.v. injections [18]. Consequently, brief high-dose i.v. infusions are being incorporated into ongoing clinical trials to evaluate the safety and efficacy of G3139 in combination with other agents (http://www.genta.com). We observed A375-RFP tumor staining after *in vivo *administration of 20 mg G4243 [carboxyfluorescein (green fluorescence)-labeled G3139]/kg (i.v.). Green fluorescence accumulated within the tumor cells thus showing that in vivo administered bcl-2-AS oligonucleotides crossed the tumor cell plasma membrane (see Additional file 1 Figure S1A).

We found that: a) G3139 reduced Bcl-2 expression in A375 tumors but had no significant effect in different non-tumor tissues (including liver, brain, lung, heart, kidney, or skeletal muscle; although, as an example, only data for the liver are shown); b) G3622 (reversed sequence), as compared to physiological saline-treated controls, does not deplete significantly Bcl-2 neither in the tumor nor in non-tumor tissues (see Additional file 1 Figure S1B). The lack of significant reduction of murine Bcl-2 expression in non-tumor tissues could be consistent with the presence of two mismatches between the human bcl-2 sequence targeted by G3139 and the murine *bcl-2 *sequence. Nevertheless, G4244 (the murine *bcl*-2-AS from Genta Inc.) was found to deplete Bcl-2 in tumor but not in non-tumor tissues in the murine B16-F10 melanoma model [11].

### Tumor GSH depletion *in vivo*

Resistance to chemo- and radiotherapy frequently associates with high GSH content, and GSH depletion can restore sensitivity to radiotherapy [19-21]. Depletion of Bcl-2 has been confirmed to decrease intracellular GSH [22]. As shown in the B16 melanoma model, selective GSH depletion in tumor cells can be achieved by combining *in vivo *administration of G3139 [increases GSH efflux through cystic fibrosis transmembrane conductance regulator (CFTR); a multidrug resistance protein 1 (MRP1)-like member of the ABC family of transport proteins], verapamil (VRP, accelerates the loss of GSH by activation of MRP1), and acivicin [ACV, blocks γ-glutamyl transpeptidase (γ-GT) and prevents recycling of L-cysteine from the extracellular pool of GSH] [11]. Thus, as part of a general chemotherapy approach, we assayed this strategy in xenografted A375 human cells.

GSH efflux in control 48-h cultured A375 cells was 1.5 ± 0.3 nmol/10^6 ^cells × h (n = 5), whereas GSH efflux in A375 cells cultured × 48 h in the presence of 2 μM G3139 and 1 μg cytofectin/ml was 3.5 ± 0.5 nmol/10^6 ^cells × h (n = 6, P < 0.01) (Bcl-2 levels in control and G3139-treated cultured cells were 28 ± 3 and 3 ± 1 units/mg protein, respectively). GSH efflux in A375 cells cultured × 48 h in the presence of 10 μM VRP was 2.5 ± 0.4 nmol/10^6 ^cells × h (n = 7, P < 0.01). γ-GT activity was 30 ± 5 and 3 ± 1 mU/10^6 ^cells in 48-h cultured A375 cells incubated, respectively, in the absence or in the presence of 1 μM ACV (n = 6, P < 0.01). *In vivo *administration of G3139, VRP, and ACV decreased GSH levels in the A375 tumor, but did not affect significantly the GSH content of different normal tissues (see Additional file 1 Table S2).

In vivo administration of G3139 + VRP + ACV decreased GSH levels in the A375 tumor, but did not affect significantly the GSH content of different normal tissues (see Additional file 1 Table S2). Single administration of any of these compounds did not decrease tumor GSH (Table S2). In the cancer cells, likely due to in vivo adaptation mechanisms, only when increasing efflux (through two different channels) associates with synthesis limitation a sharp decrease in intracellular GSH levels is obtained.

### Elimination of A375 melanoma growing *in vivo*

For conventional chemoradiotherapy paclitaxel protein-binding particles (PAC.PBP), daunorubicin (DNR), and X-rays were selected on the basis of previous *in vitro *studies, including screening most chemotherapeutic drugs used against human melanoma cells, also including vincristine, vindesine, vinblastine, bleomycin, methotrexate, arsenate, cisplatin, carmustine, dacarbazine, temozolomide, and vemurafenib (not shown). We designed a protocol based on four basic ideas: a) treatment will begin only when the tumor has reached a volume that represents a clinically relevant structure; b) fractionated and early administration of cytotoxic chemoradiotherapy, associated with Bcl-2 and GSH depletion, should kill heterogeneous melanoma cell subsets with different resistance phenotypes and may enhance uptake of G3139 into tumor cells (as it occurs with ionizing radiations [23]; c) since PAC.PBP + DNR was the best combination against A375 cells (100% tumor cell deaths *in vitro *using human MTDs; data not shown), DNR should be included at the end to eliminate possible cancer cell survivors; d) the GED, by decreasing mitochondrial GSH [24], should facilitate G3139-induced release of proapoptotic signals [10]. GED-induced depletion of tumor mitochondrial GSH was confirmed by comparing A375-RFP cells isolated from mice fed a standard diet or a GED (mitochondrial GSH levels were 8.5 ± 2.7 or 4.2 ± 1.5 nmol/10^6 ^cells, respectively, n = 5 in both cases, P < 0.05).

**Figure 3:**
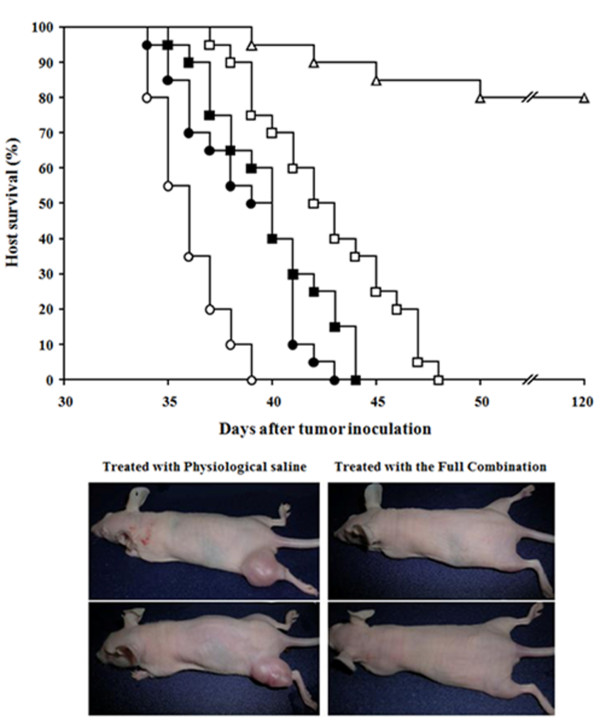
**Effect of treatment-induced regression of A375 melanoma xenografts on host survival**. A375 melanoma-bearing mice were treated as in Table 1. Host survival was studied in the following treatment conditions: (○) physiologic saline; (●) G3139 + VRP + ACV; (□) G3139 + VRP + ACV + PAC.PBP + DNR; (■) G3139 + VRP + ACV + X-rays; (Δ) G3139 + VRP + ACV + PAC.PBP + DNR + X rays. Representative photos here below correspond to two control mice treated with physiological saline and two mice treated with the full combination therapy that had > 120-day survival. Data are means + S.D. for 20 different mice in each experimental condition.

Drug doses and administration sequences (see the caption in Table 1) were evaluated in preliminary studies to optimize each element in combination-treatment protocols. One day after finishing the treatment regimen, combination of X rays + PAC.PBP + DNR had decreased tumor volume to approx. 22% of control values (treated with physiological saline) (Table 1). Addition of G3139 or G3139 + VRP + ACV further decreased tumor volume to less than 10% of controls (Table 1 one day after treatment). Nevertheless, fifteen days after the treatment, only the full combination (X rays + PAC.PBP + DNR + G3139 + VRP + ACV) induced a complete tumor regression (Table 1). Most mice (80%, Figure 3) treated with the full combination were completely cleared of tumor cells, as shown by survival beyond 120 days with no detectable tumor recurrence. The timing and order of treatment (Table 1) had significant impact on the efficacy of the regimen. If X rays were administered later (e.g. on days 11 and 12 or on days 14 and 15), or if PAC.PBP is administered later (on days 13 and 16) and DNR earlier (on days 7 and 10), the tumor volume was never below of 50 mm^3 ^in mice 1 day after receiving the full treatment (data not shown). Moreover, in mice fed a standard diet, the full treatment was unable to induce a complete tumor regression (55 ± 17 mm^3^, n = 12, 15 days after the treatment).

Complete blood cell count and standard blood chemistry were measured to evaluate the side effects of the treatment that eliminated A375 xenografts. Side effects included e.g. anemia, severe lymphopenia and neutropenia, and an increase of several tissue-damage-related enzyme activities in plasma, including aspartate aminotransferase, alanine aminotransferase, γ-glutamyl transpeptidase, alkaline phosphatase, and lactate dehydrogenase (see Additional file 1 Table S3). However, in the mice cleared of tumor (80%, > 120-day survival; Figure 3), most hematologic and clinical chemistry measures returned to normal values (untreated, non-tumor-bearing mice) 30 days after treatment (see Additional file 1 Table S3). Although the side effects measured in the treated mice are significant, such complications are commonly observed and managed in melanoma patients receiving current clinical therapies.

### Bcl-2 targeting- and chemoradiotherapy-induced elimination of A375 melanoma is not due to immune stimulation

Immune stimulation has been observed with CpG-containing antisense oligonucleotides [25]. Previous studies have shown nonantisense effects, such as production of reactive oxygen species and immunostimulatory action, elicited by *bcl*-2 antisense oligodeoxynucleotides (*bcl*-2-AS) through cytosine-phosphate-guanosine (CpG)-motifs [26]. For instance, CpG oligonucleotides may enhance the tumor response to radiotherapy [27]. Theoretically all phosphorothioate oligos, even those that do not contain CpG motifs (and G1339 has two them), will bind to TLR-9, are hence immunostimulatory to some extent, and should increase levels of circulating cytokines (some of them potentially tumoricidal) [28]. In particular, CpG oligonucleotides administered in single peritumoral subcutaneous injections three times per week resulted in elevated plasma levels of IL-12 and significant inhibition of the growth of melanoma xenografts by approx. 60% compared to the saline control [29]. Therefore it is a matter of discussion which property is the main responsible of the antitumor effects. Is the melanoma shrinkage due to the down regulation of Bcl-2 and resulting chemosensitization? Or is it due predominately to increased cytokine activity with Bcl-2 playing a lesser role *in vivo*? To answer this important question we measured leukocyte and cytokine levels in circulating blood and serum, respectively. We focused on the treatment conditions that led to complete melanoma regression *in vivo*. As shown in Table 2 the combination of G3139 + VRP + ACV + PAC.PBP + DNR + X rays (see Table 1) causes a profound leucopenia with lower levels of all white cell subtypes as compared to controls. Serum cytokine levels (including IL-1α, IL-1β, IL-2, IL-6, IL-10, IL-12, TNF-α, IFN-γ and IFN-α) were lower in A375 melanoma bearing mice treated with G3139 + VRP + ACV + PAC.PBP + DNR + X rays than in controls treated with physiological saline (Table 2). Thus it appears obvious that, in our therapeutic strategy, melanoma regression is not due to immune stimulation. In fact, immune stimulation by CpG motifs and antitumor effects in SCID mice could be independent effects [30]. As shown in Table 1 it should be noted that G3139 administration does not affect melanoma growth; and that G3139 + chemotherapy PAC.PBP + DNR only decreases melanoma growth by approx. 50% (a partial inhibition followed later by an accelerated tumor growth, not shown). Therefore, as suggested by the data in Table 1 the use of G3139, as a single agent or combined with chemotherapy, may render only limited effects against advanced melanoma.

**Table 2:** Cytokine levels in serum of A375 melanoma-bearing mice

Cytokines	Non-Tumor bearing mice	Tumor-bearing mice				
		**Days after tumor inoculation**				
		
		**7**	**11**		**17**	
		
	***Full treatment..*.**	**-**	**-**	**+**	**-**	**+**
		
**TNFα**	9 ± 3	37 ± 12**	156 ± 29**	85 ± 15**^++^	215 ± 61**	25 ± 7**^++^
**IFNα**	7 ± 2	12 ± 6	15 ± 7*	10 ± 3	17 ± 8*	6 ± 3^+^
**IFNγ**	12 ± 3	16 ± 3	17 ± 2*	13 ± 4	25 ± 7**	8 ± 4^++^
**IL-1α**	4 ± 1	15 ± 4**	20 ± 6**	12 ± 1**^+^	18 ± 4**	5 ± 1^++^
**IL-1β**	3 ± 1	8 ± 2**	7 ± 2*	5 ± 0.5*	10 ± 2**	7 ± 1**
**IL-2**	78 ± 15	116 ± 32*	200 ± 43**	84 ± 12^++^	177 ± 36**	94 ± 14^+^
**IL-6**	39 ± 14	397 ± 147**	1244 ± 296**	795 ± 206**^+^	1063 ± 253**	52 ± 15^++^
**IL-10**	86 ± 22	111 ± 39	61 ± 13	77 ± 13	93 ± 27	72 ± 18
**IL-12**	3 ± 0.5	92 ± 27**	318 ± 76**	12 ± 4**^++^	244 ± 67**	14 ± 3**^++^

Blood was collected from the tail vein every 6 h during the 24-h period of each indicated day. The days after tumor inoculation refer to those indicated in the scheme placed above the table 2. Full treatment refers to the combination of G3139 + VRP + ACV + PAC.PBP + DNR + X rays (as in table 2) that achieves a complete A375 melanoma regression. Data are mean values of the peak serum cytokine concentrations ± S.D (pg/ml) measured in 7-8 different animals. *P < 0.05, **P < 0.01 comparing for each condition versus non-tumor-bearing mice. ^+^P < 0.05, ^++^P < 0.01 comparing tumor-bearing mice receiving the full treatment (+) versus tumor-bearing mice treated with physiologic saline (-).

## Discussion

The inability of undergo apoptosis in response to chemotherapy and other external stimuli poses a selective advantage for tumor progression, metastasis formation as well as resistance to therapy in melanoma [31]. Recently numerous cellular pathways important to melanoma cell proliferation, apoptosis, or metastases have been shown to be activated. Activation occurs through specific mutations (e.g. *B-Raf*, *N-Ras*, and *PTEN*) or changes in proteins expression (e.g. PTEN, Bcl-2, NF-κB, CDK2, and cyclin D1) [32]. Thus, it is generally assumed that multi-target-directed therapies will be needed to produce significant clinical benefits in patients [32].

In particular, *bcl-2 *overexpression in different human melanoma cells appears to favour tumor progression-associated properties and *in vivo *growth [33], thus making Bcl-2 a rational target for anticancer therapy [34]. Nevertheless, as stated in the Background section, analysis of clinical melanomas suggests large variations in expression of Bcl-2 and related proteins, which possibly depend on the growth rate, the TNM staging, and/or the type of therapies applied before clinical samples were obtained. Obviously, potential adaptive changes of protein expression, under *in vivo *conditions, could be reflected by changes in the anti-death resistance potential of melanoma cells.

Studies by Stein and coworkers showed that the Bcl-2 protein profoundly affects the ability of human 518A2 melanoma cells to grow as human tumor xenografts [10]. However, *in vivo *expression or silencing of other death-related proteins was not evaluated in these studies. In fact it appears reasonable to expect that, in addition to Bcl-2, other Bcl-2-related proteins may also play growth-regulating effects in human melanoma.

We have addressed these questions in the A375 melanoma model showing that A375 cells, *in vivo*, down-regulate pro-death *bax *expression, and up-regulate anti-death *bcl*-2, *bcl-xl*, and *mcl*-1 relative to cells cultured *in vitro *(Figure 1). The impact of these changes were investigated by reversing them *in vivo *using modified A375 cells grown as xenografts (Figure 2). Forced *bax *overexpression or *mcl*-1 silencing did not affect A375 growth as compared to controls (Figure 2E). A375 melanoma cells with reduced *bcl-xl *grew *in vivo*, but more slowly than A375 controls (Figure 2E). In parallel, we found that A375 with reduced *bcl*-2 expression failed to grow after implantation and tended to regress (Figure 2E). Thus suggesting that Bcl-xl and Bcl-2 are not functionally interchangeable *in vivo*, and that Bcl-2 alone (despite down regulation of *bax *and up-regulation of *bcl-xl*) could be a relevant target in melanoma therapy.

Why would Bcl-2, which is only one of the many regulators of apoptosis, be essential for melanoma progression *in vivo *(Figure 2E)? The more aggressive behaviour of different Bcl-2 overexpressing melanomas was associated to an increase in several metalloproteases (e.g. MMP-2, MMP-7, and MT1-MMP) expression, and to an elevated microvessel density as compared to parental cells [33]. In agreement with those findings it was shown, e.g., that a) Bcl-2 promotes invasion and lung metastasis of Bcl-2 overexpressing non-small cell lung cancer cells by inducing matrix metalloproteinase-2 [35]; b) membrane type-1 matrix metalloproteinase promotes human melanoma invasion and growth [36]; and c) Bcl-2 overexpression in human melanoma cells increases angiogenesis through VEGF mRNA stabilization and HIF-1-mediated transcriptional activity [37], and regulates HIF-1alpha protein stabilization in hypoxic melanoma cells via HSP90 [38]. Nevertheless these possibilities have been questioned since: a) Bcl-2 down-regulation in 518A2 melanoma cells did not associate with down-regulation of MMP-2 or MMP-9; and b) also in 518A2 tumor cells, transfected with the Bcl-2 plasmid and growing as xenografts, extensive necrosis in the setting of very poor vascularity was observed [10]. Therefore, correlations between Bcl-2 overexpression and MMPs or angiogenesis lack experimental evidence. Besides it is known that Bcl-2 and Bcl-xl are antiproliferative by facilitating arrest at G0-G1 [38]. The dual functions in apoptosis and cell cycle are coordinately regulated by the multi-domain Bcl-2 family members and suggest that survival is maintained at the expense of proliferation [39].

Anti-*bcl*-2 antisense therapy decreases Bcl-2 in A375 tumors (Additional file 1 Figure S1B). G3139, if combined with VRP + ACV, also depletes melanoma GSH (Additional file 1 Table S2). Bcl-2 down-regulation in melanoma causes sensitization to chemoradiotherapy (Table 1). However, only combined depletion of Bcl-2 and GSH leads to a complete xenografts regression by chemoradiotherapy (Table 1), and long-term survival of the treated mice (Figure 3). Blc-2 inhibits GSH efflux and, thus, favours GSH accumulation within the cancer cell [14]. The use of G3139 facilitates GSH efflux [22]. However, unless this is combined with GSH efflux facilitation through MRP1 and a gamma-glutamyl-transpeptidase inhibitor to prevent de novo GSH synthesis, the cancer cell responds with a rebound in GSH synthesis (see e.g. [22]).

G3139 decreases Bcl-2 levels to less than 30% of control values se the legend to Table 1) but it does not inhibit tumor growth. Hence, until more effective and specific anti-Bcl-2 agents/treatments are developed, the Bcl-2/GSH double targeting in combination with chemoradiotherapy appears a reasonable, clinically feasible, and effective approach.

A phase III trial designed to confirm safety and efficacy results of G3139 combined with dacarbazine in melanoma patients (not previously receiving chemotherapy), indicated that G3139 has an acceptable clinical safety profile [40], although positive impact on different clinical end points, including progression-free survival and overall survival, has not been obtained (see http://www.genta.com). Thus indicating that G3139 + dacarbazine is not the right strategy, as suggested by our data (Table 1). Nevertheless standard chemoradiotherapy, and additional drugs (GED, VRP, and ACV), have known clinical applications and its use in melanoma has been recently discussed [11].

Optimization of chemoradiotherapy and antisense therapy targeting *bcl*-2 (Table 1) simplifies the treatment that eliminated metastatic B16 melanoma from the mouse liver, since avoids the need of TNF-α, and IFN-γ administration (and their side effects). Thus, our strategy likely appears more patient**-**friendly and effective (Figure 3). Moreover, our combination therapy appears easy to standardized since effective doses in human melanoma-bearing mice are within clinically acceptable and tolerated ranges.

Finally it is important to remark that recently developed target therapies for the treatment of late-stage melanoma were also considered for our combination strategy. This including vemurafenib (a B-Raf enzyme inhibitor also known as PLX4032) [41] and ipilimumab (a fully human antibody that binds to cytotoxic T lymphocyte-associated inhibitor, which may in turn augment T-cell responses to melanoma cells) [42]. B-Raf knockdown leads to apoptosis in the melanoma cell line A375 [43], however it did not come up as the best option on the basis of our preliminary *in vitro *studies (see under Results). On the other hand ipilimumab may be very useful if applied after our immunosuppressive combination therapy, during the recovery phase, were in some cases a few malignant melanoma cells may have managed to survive.

## Conclusions

Our results suggest that Bcl-2 may be an important target against melanoma progression and resistance to therapy, not only for its antiapoptotic role but also for regulating GSH levels and, likely, for other unrevealed mechanisms. Bcl-2 appears to play a prevalent role even when other Bcl-2 family protein are also overexpressed. However *in vivo bcl*-2-AS administration-induced Bcl-2 depletion, by itself, does not inhibit tumor growth. Even the simultaneous depletion of GSH and Bcl-2 is uneffective unless chemoradiotherapy is also applied. Nevertheless, before considering practical applications, the importance of Bcl-2 and the mechanisms involved must be investigated in a sufficiently large number of human melanomas showing different rates of expression of Bcl-2 and Bcl-2-related proteins. If Bcl-2 and/or other(s) related protein(s) is/are identified as key target(s), our strategy (or variations of it) may improve the poor prognosis of advance melanoma-bearing patients.

## Abbreviations

GSH: glutathione; bcl-2-AS: bcl-2 antisense oligodeoxynucleotides; CpG-motifs: cytosine-phosphate-guanosine-motifs; DMEM: Dulbecco's modified Eagle's medium; RFP: red fluorescent protein; PEI: polyethylenimine; GED: L-glutamine-enriched diet; CFTR: cystic fibrosis transmembrane conductance regulator; MRP1: multidrug resistance protein 1; VRP: verapamil; ACV: acivicin; γ -GT: γ-glutamyl transpeptidase; PAC.PBP: Paclitaxel protein-binding particles; DNR: daunorubicin; MMPs: metalloproteases.

## Competing interests

The authors declare that they have no competing interests.

## Authors' contributions

SM carried out cell culture and transfections, RT-PCR experiments, and participated in animal treatments. MLR worked on western blots. AO performed isolation and compartmentation of tumor cells. SP worked on gene transfer and enzyme immunoassays. EO carried out tumor xenografts and participated in animal treatments. MA performed GSH measurements and in vivo toxicity studies. IP worked on animal irradiations. MC carried out laser microdissections. BDB planned the studies and revised the manuscript. JME participated in animal treatments, planned the studies, and wrote the manuscript. All authors have read and approved the final manuscript.

## Supplementary Material

Additional file 1 **Figure S1: *In vivo *G3139 uptake and Bcl-2 depletion in A375 melanoma cells**. (A) ***In vivo ***distribution of ***blc***-2-AS in RFP-expressing A375 tumors growing in mice treated with G4243 (20 mg/kg): transmission, red and green fluorescence, and nuclei which were labeled with Hoescht 33342 (Sigma) [11]. Microscopic examination (A) was performed 24 h after G4243 administration, whereas Bcl-2 levels (B) were measured 24, and 48 h after G4243 administration. Human and murine Bcl-2 were quantified by enzyme immunoassay (see Methods) in A375 and murine tissue samples, respectively, obtained after treating xenografted mice (7 days after A375 inoculation) with physiological saline or 20 mg G3622 or G3139 (***bcl***-2-AS)/kg. Histological examination showed that in tumor tissue samples the highest % of tissue mass (> 92% in all cases) corresponds to A375 cells. Bcl-2 levels, measured as units/mg protein remained < 30% of control values in all mice treated with G3139 alone or in combination with the other treatments displayed in Table 1 (data not shown). Bars are means ± S.D. of 4-5 different experiments, *P < 0.01 (comparing each ***bcl***-2-AS versus controls). Treatment with G3139 or G3622 did not affect significantly Bcl-2 levels in brain, lung, heart, kidney, or skeletal muscle relative to untreated controls (data not shown). **Table S1: Expression of bax, bcl-2, bcl-xl, and mcl-1 in A375 control xenografts or in tumors of mice inoculated with A375/Tet-bax, A375/bcl-xl-shRNA, A375/bcl-2-shRNA, or A375/mcl-1-shRNA cells**. A375-RFP cells were isolated by laser microdissection (as indicated under Methods) 14 days after tumor inoculation. The data, expressing fold change (quantitative RT-PCR, see under Methods for calculations), show mean values ± S.D. for 4 different experiments (*p < 0.05 for the genes displayed comparing control A375-RFP cells and their different variants, isolated from ***in vivo ***growing tumors, versus 24 h-cultured A375-RFP cells; +p < 0.05 for all genes displayed comparing control A375-RFP cells versus their different variants).Values obtained in A375-RFP cells transfected with the negative control plasmid vector HK were not significantly different from those obtained in controls (not shown). **Table S2: GSH content in tumor cells and tissues from A375 melanoma xenograft-bearing mice treated with G3139, VRP, and/or ACV**. The 10 days combination therapy schedule is shown. G3139 (20 mg/kg each 72 h, i.v.), VRP (1 mg/kg × day, i.p.), ACV (2 mg/kg × day, i.p.) were given starting 1 week after inoculation of A375 cells. Tumor and tissue samples were obtained 24 h after finishing the treatment period. For details of sample processing see [11]. Data are means ± S.D. for n = 8-9 mice in each condition. Values are mmol/g of tissue, except in A375 cells for which GSH is expressed as nmol/10**^6 ^**cells. *p < 0.01 comparing different treatment conditions versus physiological saline (PS)-treated control mice; **^+^**p < 0.01 comparing tumor-bearing mice versus non-tumor-bearing mice. GSH content in brain, lung, heart, glandular stomach, skeletal muscle, bone marrow, ovary, and erythrocytes was not affected by the growing tumor or by any of the treatments used (not shown). **Table S3: Hematology and clinical chemistry data in A375 melanoma-bearing mice treated to induce tumor suppression**. Standard cell count and chemistry were measured in peripheral blood samples taken from the saphenous vein. Full treatment means the combination of G3139 + verapamil + acivicin + paclitaxel protein-binding particles + daunorubicin + X-rays (given as indicated in the caption of Table 2). Tumor-bearing mice were sacrificed 1 or 30 days after finishing the full treatment, whereas controls treated with physiological saline were sacrificed 1 day after finishing the treatment. Data are means ± S.D. for 8-9 different mice in each experimental condition. *p < 0.05 comparing tumor-bearing mice versus non-tumor-bearing mice; **^+^**p < 0.05 comparing full treatment versus treatment with physiological saline. Click here for file

## References

[B1] Bhatia S Tykodi SS Thompson JA Treatment of metastatic melanoma: an overview Oncology (Williston Park) 2009 23 488 496 PMC2737459 19544689

[B2] Rass K Tilgen W Treatment of melanoma and nonmelanoma skin cancer Adv Exp Med Biol 2008 624 296 318 10.1007/978-0-387-77574-6_23 18348465

[B3] Reed JC Bcl-2 family proteins: strategies for overcoming chemoresistance in cancer Adv Pharmacol 1997 41 501 532 9204157 10.1016/s1054-3589(08)61070-4

[B4] Kim R Emi M Tanabe K Toge T Therapeutic potential of antisense Bcl-2 as a chemosensitizer for cancer therapy Cancer 2004 101 2491 2502 10.1002/cncr.20696 15503311

[B5] Eberle J Kurbanov BM Hossini AM Trefzer U Fecker LF Overcoming apoptosis deficiency of melanoma-hope for new therapeutic approaches Drug Resist Updat 2007 10 218 234 10.1016/j.drup.2007.09.001 18054518

[B6] Pattingre S Tassa A Qu X Garuti R Liang XH Mizushima N Packer M Schneider MD Levine B Bcl-2 antiapoptotic proteins inhibit Beclin 1-dependent autophagy Cell 2005 122 927 939 10.1016/j.cell.2005.07.002 16179260

[B7] Bush JA Li G The role of Bcl-2 family members in the progression of cutaneous melanoma Clin Exp Metastasis 2003 20 531 539 10.1023/A:1025874502181 14598887

[B8] Eberle J Hossini AM Expression and function of bcl-2 proteins in melanoma Curr Genomics 2008 9 409 419 10.2174/138920208785699571 19506730 PMC2691663

[B9] Placzek WJ Wei J Kitada S Zhai D Reed JC Pellecchia M A survey of the anti-apoptotic Bcl-2 subfamily expression in cancer types provides a platform to predict the efficacy of Bcl-2 antagonists in cancer therapy Cell Death Dis 2010 1 e40 10.1038/cddis.2010.18 21364647 PMC3032312

[B10] Benimetskaya L Ayyanar K Kornblum N Castanotto D Rossi J Wu S Lai J Brown BD Popova N Miller P Bcl-2 protein in 518A2 melanoma cells in vivo and in vitro Clin Cancer Res 2006 12 4940 4948 10.1158/1078-0432.CCR-06-1002 16914583

[B11] Mena S Benlloch M Ortega A Carretero J Obrador E Asensi M Petschen I Brown BD Estrela JM Bcl-2 and glutathione depletion sensitizes B16 melanoma to combination therapy and eliminates metastatic disease Clin Cancer Res 2007 13 2658 2666 10.1158/1078-0432.CCR-06-2642 17473197

[B12] Paine-Murrieta GD Taylor CW Curtis RA Lopez MH Dorr RT Johnson CS Funk CY Thompson F Hersh EM Human tumor models in the severe combined immune deficient (scid) mouse Cancer Chemother Pharmacol 1997 40 209 214 10.1007/s002800050648 9219503

[B13] Ortega AL Carretero J Obrador E Gambini J Asensi M Rodilla V Estrela JM Tumor cytotoxicity by endothelial cells. Impairment of the mitochondrial system for glutathione uptake in mouse B16 melanoma cells that survive after in vitro interaction with the hepatic sinusoidal endothelium J Biol Chem 2003 278 13888 97 10.1074/jbc.M207140200 12578841

[B14] Ortega A Ferrer P Carretero J Obrador E Asensi M Pellicer JA Estrela JM Down-regulation of glutathione and Bcl-2 synthesis in mouse B16 melanoma cells avoids their survival during interaction with the vascular endothelium J Biol Chem 2003 278 39591 9 10.1074/jbc.M303753200 12881529

[B15] Bradford MM A rapid and sensitive method for the quantitation of microgram quantities of protein utilizing the principle of protein-dye binding Anal Biochem 1976 72 248 254 10.1016/0003-2697(76)90527-3 942051

[B16] New LS Chan EC Evaluation of BEH C18, BEH HILIC, and HSS T3 (C18) column chemistries for the UPLC-MS-MS analysis of glutathione, glutathione disulfide, and ophthalmic acid in mouse liver and human plasma J Chromatogr Sci 2008 46 209 214 18334086 10.1093/chromsci/46.3.209

[B17] Asensi M Sastre J Pallardo FV Garcia de la Asuncion J Estrela JM Vina J A high-performance liquid chromatography method for measurement of oxidized glutathione in biological samples Anal Biochem 1994 217 323 328 10.1006/abio.1994.1126 8203763

[B18] Brown BD Paine-Murietta GD Julian TN Warrell RP Brief intravenous infusions of oblimersen (Genasense; Bcl-2 antisense) alone and in combination with multiple agents are highly effective in human tumor xenografts J Clin Oncol 2007 18S June 20 Supplement 14061 2007 ASCO Annual Meeting Proceedings Part I 25

[B19] Anasagasti MJ Martin JJ Mendoza L Obrador E Estrela JM McCuskey RS Vidal-Vanaclocha F Glutathione protects metastatic melanoma cells against oxidative stress in the murine hepatic microvasculature Hepatology 1998 27 1249 1256 10.1002/hep.510270510 9581678

[B20] Estrela JM Obrador E Navarro J Lasso De la Vega MC Pellicer JA Elimination of Ehrlich tumours by ATP-induced growth inhibition, glutathione depletion and X-rays Nat Med 1995 1 84 88 10.1038/nm0195-84 7584960

[B21] Estrela JM Ortega A Obrador E Glutathione in cancer biology and therapy Crit Rev Clin Lab Sci 2006 43 143 181 10.1080/10408360500523878 16517421

[B22] Benlloch M Ortega A Ferrer P Segarra R Obrador E Asensi M Carretero J Estrela JM Acceleration of glutathione efflux and inhibition of gamma-glutamyltranspeptidase sensitize metastatic B16 melanoma cells to endothelium-induced cytotoxicity J Biol Chem 2005 280 6950 6959 10.1074/jbc.M408531200 15561710

[B23] Anai S Brown BD Nakamura K Goodison S Hirao Y Rosser CJ Irradiation of human prostate cancer cells increases uptake of antisense oligodeoxynucleotide Int J Radiat Oncol Biol Phys 2007 68 1161 1168 10.1016/j.ijrobp.2007.03.058 17637391

[B24] Benlloch M Mena S Ferrer P Obrador E Asensi M Pellicer JA Carretero J Ortega A Estrela JM Bcl-2 and Mn-SOD antisense oligodeoxynucleotides and a glutamine-enriched diet facilitate elimination of highly resistant B16 melanoma cells by tumor necrosis factor-alpha and chemotherapy J Biol Chem 2006 281 69 79 16263711 10.1074/jbc.M507471200

[B25] Vollmer J Progress in drug development of immunostimulatory CpG oligodeoxynucleotide ligands for TLR9 Expert Opin Biol Ther 2005 5 673 682 10.1517/14712598.5.5.673 15934842

[B26] Kim R Emi M Matsuura K Tanabe K Antisense and nonantisense effects of antisense Bcl-2 on multiple roles of Bcl-2 as a chemosensitizer in cancer therapy Cancer Gene Ther 2007 14 1 11 10.1038/sj.cgt.7700986 17041564

[B27] Zha L Qiao T Yuan S Lei L Enhancement of radiosensitivity by CpG-oligodeoxyribonucleotide-7909 in human non-small cell lung cancer A549 cells Cancer Biother Radiopharm 2010 25 165 170 10.1089/cbr.2009.0686 20423229

[B28] Klinman DM Currie D Gursel I Verthelyi D Use of CpG oligodeoxynucleotides as immune adjuvants Immunol Rev 2004 199 201 216 10.1111/j.0105-2896.2004.00148.x 15233736

[B29] Krepler C Wacheck V Strommer S Hartmann G Polterauer P Wolff K Pehamberger H Jansen B CpG oligonucleotides elicit antitumor responses in a human melanoma NOD/SCID xenotransplantation model J Invest Dermatol 2004 122 387 391 10.1046/j.0022-202X.2004.22202.x 15009720

[B30] Wacheck V Krepler C Strommer S Heere-Ress E Klem R Pehamberger H Eichler HG Jansen B Antitumor effect of G3139 Bcl-2 antisense oligonucleotide is independent of its immune stimulation by CpG motifs in SCID mice Antisense Nucleic Acid Drug Dev 2002 12 359 367 10.1089/108729002321082438 12568310

[B31] Smalley KS Herlyn M Towards the targeted therapy of melanoma Mini Rev Med Chem 2006 6 387 393 10.2174/138955706776361402 16613575

[B32] Sosman JA Puzanov I Molecular targets in melanoma from angiogenesis to apoptosis Clin Cancer Res 2006 12 2376s 2383s 10.1158/1078-0432.CCR-05-2558 16609062

[B33] Trisciuoglio D Desideri M Ciuffreda L Mottolese M Ribatti D Vacca A Del Rosso M Marcocci L Zupi G Del Bufalo D Bcl-2 overexpression in melanoma cells increases tumor progression-associated properties and in vivo tumor growth J Cell Physiol 2005 205 414 421 10.1002/jcp.20413 15920759

[B34] Piro LD Apoptosis, Bcl-2 antisense, and cancer therapy Oncology (Williston Park) 2004 18 5 10 15651171

[B35] Choi J Choi K Benveniste EN Rho SB Hong YS Lee JH Kim J Park K Bcl-2 promotes invasion and lung metastasis by inducing matrix metalloproteinase-2 Cancer Res 2005 65 5554 5560 10.1158/0008-5472.CAN-04-4570 15994927

[B36] Iida J Wilhelmson KL Price MA Wilson CM Pei D Furcht LT Carthy JB Membrane type-1 matrix metalloproteinase promotes human melanoma invasion and growth J Invest Dermatol 2004 122 167 176 10.1046/j.0022-202X.2003.22114.x 14962105

[B37] Iervolino A Trisciuoglio D Ribatti D Candiloro A Biroccio A Zupi G Del Bufalo D Bcl-2 overexpression in human melanoma cells increases angiogenesis through VEGF mRNA stabilization and HIF-1-mediated transcriptional activity FASEB J 2002 16 1453 1455 12205045 10.1096/fj.02-0122fje

[B38] Trisciuoglio D Gabellini C Desideri M Ziparo E Zupi G Del Bufalo D Bcl-2 regulates HIF-1alpha protein stabilization in hypoxic melanoma cells via the molecular chaperone HSP90 PLoS One 2010 5 e11772 10.1371/journal.pone.0011772 20668552 PMC2910721

[B39] Zinkel S Gross A Yang E BCL2 family in DNA damage and cell cycle control Cell Death Differ 2006 13 1351 1359 10.1038/sj.cdd.4401987 16763616

[B40] Bedikian AY Millward M Pehamberger H Conry R Gore M Trefzer U Pavlick AC DeConti R Hersh EM Hersey P Bcl-2 antisense (oblimersen sodium) plus dacarbazine in patients with advanced melanoma: the Oblimersen Melanoma Study Group J Clin Oncol 2006 24 4738 4745 10.1200/JCO.2006.06.0483 16966688

[B41] Smalley KS PLX-4032, a small-molecule B-Raf inhibitor for the potential treatment of malignant melanoma Curr Opin Investig Drugs 2010 11 699 706 20496265

[B42] Cameron F Whiteside G Perry C Ipilimumab: first global approval Drugs 2011 71 1093 1104 10.2165/11594010-000000000-00000 21668044

[B43] Sala E Mologni L Truffa S Gaetano C Bollag GE Gambacorti-Passerini C BRAF silencing by short hairpin RNA or chemical blockade by PLX4032 leads to different responses in melanoma and thyroid carcinoma cells Mol Cancer Res 2008 6 751 759 10.1158/1541-7786.MCR-07-2001 18458053

