# Supplementary material for: Glutathione and Bcl-2 targeting facilitates elimination by chemoradiotherapy of human A375 melanoma xenografts overexpressing bcl-xl, bcl-2, and mcl-1
Source: J Transl Med. 2012 Jan 10;10:8. doi: 10.1186/1479-5876-10-8 (PMC3268086; doi:10.1186/1479-5876-10-8)
Supplement: Additional file 1 — Figure S1: In vivo G3139 uptake and Bcl-2 depletion in A375 melanoma cells. (A) In vivo distribution of blc-2-AS in RFP-expressing A375 tumors growing in mice treated with G4243 (20 mg/kg): transmission, red and green fluorescence, and nuclei which were labeled with Hoescht 33342 (Sigma) [11]. Microscopic examination (A) was performed 24 h after G4243 administration, whereas Bcl-2 levels (B) were measured 24, and 48 h after G4243 administration. Human and murine Bcl-2 were quantified by enzyme immunoassay (see Methods) in A375 and murine tissue samples, respectively, obtained after treating xenografted mice (7 days after A375 inoculation) with physiological saline or 20 mg G3622 or G3139 (bcl-2-AS)/kg. Histological examination showed that in tumor tissue samples the highest % of tissue mass (> 92% in all cases) corresponds to A375 cells. Bcl-2 levels, measured as units/mg protein remained < 30% of control values in all mice treated with G3139 alone or in combination with the other treatments displayed in Table 1 (data not shown). Bars are means ± S.D. of 4-5 different experiments, *P < 0.01 (comparing each bcl-2-AS versus controls). Treatment with G3139 or G3622 did not affect significantly Bcl-2 levels in brain, lung, heart, kidney, or skeletal muscle relative to untreated controls (data not shown). Table S1: Expression of bax, bcl-2, bcl-xl, and mcl-1 in A375 control xenografts or in tumors of mice inoculated with A375/Tet-bax, A375/bcl-xl-shRNA, A375/bcl-2-shRNA, or A375/mcl-1-shRNA cells. A375-RFP cells were isolated by laser microdissection (as indicated under Methods) 14 days after tumor inoculation. The data, expressing fold change (quantitative RT-PCR, see under Methods for calculations), show mean values ± S.D. for 4 different experiments (*p < 0.05 for the genes displayed comparing control A375-RFP cells and their different variants, isolated from in vivo growing tumors, versus 24 h-cultured A375-RFP cells; +p < 0.05 for all genes displayed comparing contr [file 1479-5876-10-8-S1.DOC]

**Figure S1**


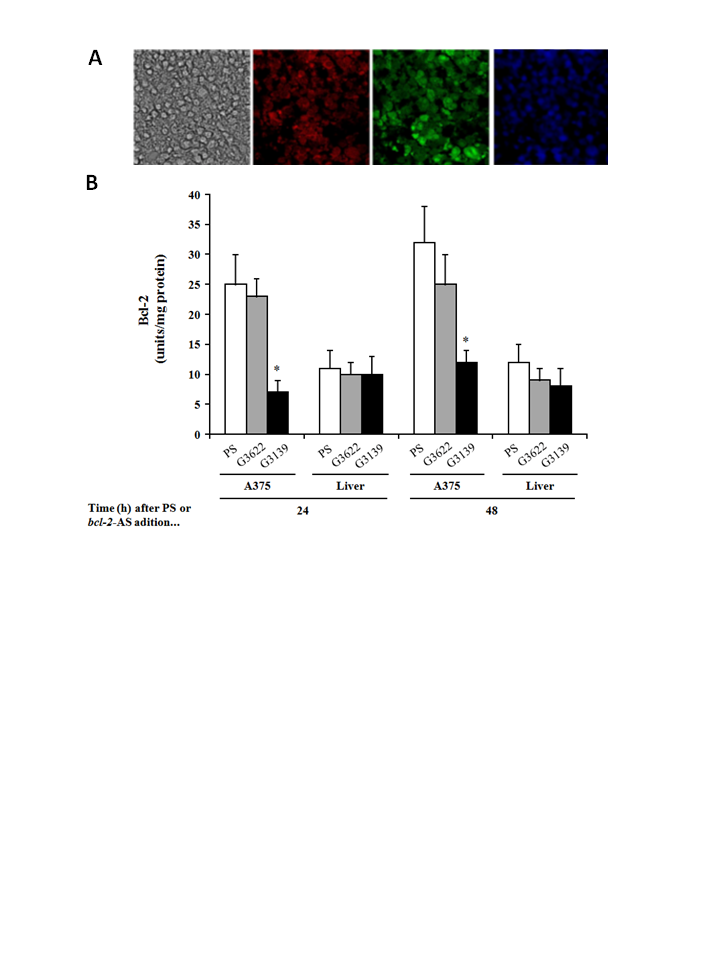

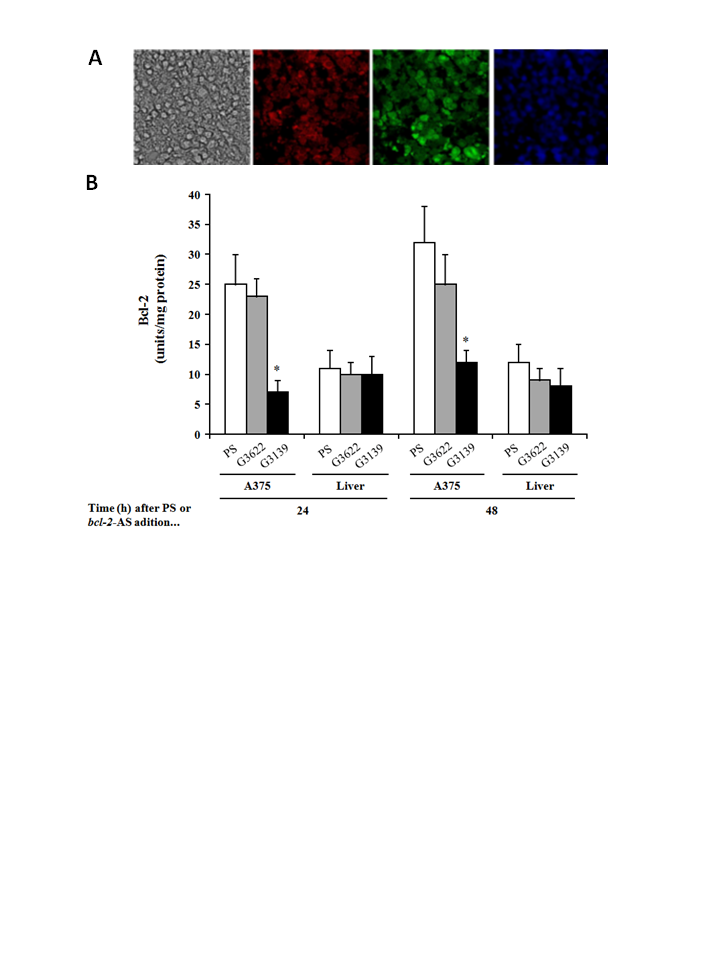


**Table S1**

|  |  | **Genes** |  |  |  |
| --- | --- | --- | --- | --- | --- |
| **A375-RFP cells inoculated** | ***bax*** | ***bcl-xl*** | ***bcl*-2** | ***mcl*-1** | |
| **Control** | -2.0 ± 0.4^*^ | 2.5 ± 0.5^*^ | 2.9 ± 0.4^*^ | 1.5 ± 0.3^*^ | |
| **Tet-*bax*** | 4.3 ± 0.4^*^ | 2.2 ± 0.3^*^ | 2.7 ± 0.3^*^ | 1.4 ± 0.3^*^ | |
| ***bcl-xl*-shRNA** | -2.2 ± 0.3^*^ | 0.1 ± 0.05^*^ | 3.5 ± 0.3^*^ | 1.7 ± 0.2^*^ | |
| ***bcl-*2-shRNA** | -1.9 ± 0.2^*^ | 2.9 ± 0.4^*^ | 0.1 ± 0.05^*^ | 1.2 ± 0.2 | |
| ***mcl*-1-shRNA** | -1.8 ± 0.3^*^ | 2.4 ± 0.4^*^ | 3.4 ± 0.5^*^ | 0.2 ± 0.1^*^ | |

**Table S2**

|  | **A375** | **Liver** | **Kidney** |
| --- | --- | --- | --- |
| **Non-Tumor-bearing mice** |  | 6.5 ± 0.4 | 2.6 ± 0.3 |
| **Tumor-bearing mice** |  |  |  |
| **+ PS** | 14 ± 3 | 3.8 ± 0.3^+^ | 1.7 ± 0.3^+^ |
| **+ G3139** | 16 ± 4 | 3.9 ± 0.5^+^ | 1.7 ± 0.2^+^ |
| **+ VRP** | 18 ± 3 | 3.7 ± 0.4 | 1.8 ± 0.2^+^ |
| **+ ACV** | 16 ± 3 | 4.3 ± 0.5 | 2.2 ± 0.4 |
| **+ G3139 + VRP** | 27 ± 3^*^ | 4.2 ± 0.2^+^ | 2.0 ± 0.3^+^ |
| **+ G3139 + ACV** | 10 ± 2 | 3.5 ± 0.4 | 1.9 ± 0.2^+^ |
| **+ G3139 + VRP + ACV** | 5 ± 2^*^ | 4.0 ± 0.5^+^ | 2.1 ± 0.1^+^ |

**Table S3**

|  | | | |  | | **Non-tumor-bearing mice** |  | **Tumor-bearing mice** | | | | | | | | |  |
| --- | --- | --- | --- | --- | --- | --- | --- | --- | --- | --- | --- | --- | --- | --- | --- | --- | --- |
|  | | | |  | |  |  | **+Physiologic saline** |  | |  | **+Full treatment** |  | |  | **30 days after**  **full treatment** |  |
| **Hematology** |  | | |  | |  |  | | |  | | | |  | | | |
| **Hematocrit (%)** | | | |  | | 38.2 ± 1.0 | 30.2 ± 2.1^*^  21.8 ± 3.7^*+^ 32.9 ± 2.4^*^ | | | | | | | | | | |
| **Hemoglobin (g/dl)** | | | |  | | 12.9 ± 0.5 | 12.0 ± 0.4 7.5 ± 0.9^*+^ 12.0 ± 0.6 | | | | | | | | | | |
| **Erythrocites (10^6^/μl)** | | | |  | | 8.7 ± 0.3 | 6.1 ± 0.4^*^  4.4 ± 0.7^*+^ 7.9 ± 0.4^*+^ | | | | | | | | | | |
| **Platelets (10^3^/μl)** | | | |  | | 410 ± 38 | 336 ± 32^*^    111 ± 25^*+^ 379 ± 57 | | | | | | | | | | |
| **Leukocytes (10^3^/μl)** | | | |  | | 2.7 ± 0.4 | 2.1 ± 0.4 0.5 ± 0.2^*+^ 2.2 ± 0.5 | | | | | | | | | | |
| **Neutrophils (10^3^/μl)** | | | |  | | 1.0 ± 0.2 | 0.8 ± 0.1 0.2 ± 0.05^*+^ 1.0 ± 0.3 | | | | | | | | | | |
| **Lymphocytes (10^3^/μl)** | | | |  | | 1.1 ± 0.3 | 1.0 ± 0.2 0.2 ± 0.1^*+^ 1.0 ± 0.2 | | | | | | | | | | |
| **Monocytes (10^3^/μl)** | | | |  | | 0.1 ± 0.03 | 0.05 ± 0.01^*^  0.01 ± 0.005^*+^  0.1 ± 0.05 | | | | | | | | | | |
| **Eosinophils (10^3^/μl)** | | | |  | | 0.1 ± 0.05 | 0.05 ± 0.01 0.01 ± 0.004^*+^ 0.1 ± 0.03 | | | | | | | | | | |
| **Basophils (10^3^/μl)** | | | |  | | 0.0 ± 0.0 | 0.0 ± 0.0 0.0 ± 0.0 0.0 ± 0.0 | | | | | | | | | | |
| **Clinical chemistry** | |  | |  | |  |  | | |  | | | |  | | | |
| **Urea (mg/dl)** | | | |  | | 45.6 ± 2.5 | 53.1 ± 3.8^*^ 60.2 ± 2.7^*+^ 50.2 ± 3.3 | | | | | | | | | | |
| **Uric acid (mg/dl)** | | | |  | | 2.2 ± 0.2 | 1.7 ± 0.3 0.6 ± 0.3^*+^   2.0 ± 0.3 | | | | | | | | | | |
| **Total protein (g/dl)** | | | |  | | 4.4 ± 0.5 | 3.9 ± 0.3 3.8 ± 0.2 4.1 ± 0.2 | | | | | | | | | | |
| **Albumin (g/dl)** | | | |  | | 3.1 ± 0.2 | 2.7 ± 0.3 2.5 ± 0.4^*^ 2.9 ± 0.2 | | | | | | | | | | |
| **Creatinin (mg/dl)** | | | |  | | 0.5 ± 0.2 | 0.5 ± 0.07 0.7 ± 0.05 0.5 ± 0.2 | | | | | | | | | | |
| **Glucose (mg/dl)** | | | |  | | 143 ± 19 | 158 ± 21 103 ± 17^* +^  155 ± 25 | | | | | | | | | | |
| **Total bilirubin (mg/dl)** | | | |  | | 0.5 ± 0.1 | 0.4 ± 0.05 0.8 ± 0.2^*+^ 0.5 ± 0.2 | | | | | | | | | | |
| **Direct bilirubin (mg/dl)** | | | |  | | 0.1 ± 0.04 | 0.05 ± 0.01^*^ 0.2 ± 0.08^*+^  0.1 ± 0.03 | | | | | | | | | | |
| **Aspartate aminotransferase (IU/l)** | | | | | | 144 ± 36 | 227 ± 58^*^  649 ± 88^*+^ 195 ± 48 | | | | | | | | | | |
| **Alanine aminotransferase (IU/l)** | | |  | | | 7.2 ± 2.4 | 59.7 ± 12.3^*^  259 ± 38^*+^  42.7 ± 10.8^*^ | | | | | | | | | | |
| **Gamma-glutamyl transpeptidase (IU/l)**  2.6 ± 0.4 | | | | | | | 5.8 ± 1.1^*^    29.3 ± 6.4^*+^  4.5 ± 1.7^*^ | | | | | | | | | | |
| **Alkaline phosphatase (IU/l)** | | |  | | 123 ± 15 | | 128 ± 39  412 ± 53^*+^ 155 ± 42 | | | | | | | | | | |
| **Lactate dehydrogenase (IU/l)** | | |  | | 225 ± 42 | | 377 ± 58^*^  946 ± 258^*+^ 349 ± 67^*^ | | | | | | | | | | |
